# Perceptual load modulates contour integration in conscious and unconscious states

**DOI:** 10.7717/peerj.7550

**Published:** 2019-08-22

**Authors:** Kaiwen Cheng, Keyu Yang, Long Qin, Yixuan Zhuo, Hongmei Yan

**Affiliations:** 1 MOE Key Lab for Neuroinformation, University of Electronic Science and Technology of China, Chengdu, Sichuan, China; 2 School of Foreign Languages, Southwest Jiaotong University, Chengdu, Sichuan, China

**Keywords:** Contour integration, Perceptual load, Topological property, Saliency, Conscious awareness

## Abstract

Previous research has documented that contour detection and integration may either be affected by local features such as the distances between elements or by high-level cognitive factors such as attention in our visual system. Less is known about how low and high level factors interact to influence contour integration. In this paper, we investigated how attention modulates contour integration through saliency (different element spacing) and topological propert ies (circle or S-shaped) when the state of conscious awareness is manipulated. A modified inattentional blindness (IB) combined with the Posner cuing paradigm was adopted in our three-phased experiment (unconscious-training-conscious). Attention was manipulated with high or low perceptual load for a foveal go/no-go task. Cuing effects were utilized to assess the covert processing of contours prior to a peripheral orientation discrimination task. We found that (1) salient circles and S-contours induced different cuing effects under low perceptual load but not with high load; (2) no consistent pattern of cuing effects was found for non-salient contours in all the conditions; (3) a positive cuing effect was observed for salient circles either consciously or unconsciously while a negative cuing effect occurred for salient S-contours only consciously. These results suggest that conscious awareness plays a pivotal role in coordinating a closure effect with the level of perceptual load. Only salient circles can be successfully integrated in an unconscious state under low perceptual load although both salient circles and S-contours can be done consciously. Our findings support a bi-directional mechanism that low-level sensory features interact with high-level cognitive factors in contour integration.

## Introduction

Contour detection and integration are crucial for the visual system to generate a coherent representation of visual objects, especially in complex environments. Visual contour integration is assumed to vary with low-level features such as color (Mcilhagga & Mullen, 1996; Mullen, Beaudot & Mcilhagga, 2000), orientation (Nelson & Frost, 1985), spatial frequency (Field, Hayes & Hess, 1993), saliency (Li & Gilbert, 2002), and topology (Kovács & Julesz, 1993). For example, the space between adjacent contour segments, which is an index to define saliency, is an important low level factor that influences the performance of contour detection (Strother & Alferov, 2014). Contour integration generally declines with increasing spaces between neighboring elements (Field, Hayes & Hess, 1993; Strother & Alferov, 2014). Global contour saliency is based on the local integration mechanisms of intermediate spatial extent (Li & Gilbert, 2002). Kovács & Julesz (1993) found that the visual system was more sensitive to closed contours in contrast to open contours and attributed the sensibility to a probability computation based on fragments or local features of a contour (bottom-up). Tversky, Geisler & Perry (2004) reported that circles were slightly more detectable than S-contours even if two types contained the same number of elements at the same local patch curvature. The authors suggested that a local mechanism or the interaction of local continuation (i.e., collinearity) and proximity (i.e., distances) mechanisms could induce global perceptual wholes. It is widely accepted that proximity and continuation can be implemented physiologically through long-range horizontal connections in the primary visual cortex (V1) (Gilbert & Li, 2013; Hess, Hayes & Field, 2003; Li & Gilbert, 2002; Li, 1997).

However, Gerhardstein et al. (2012) argued that a local mechanism did not fully account for the detection advantage of closed over open contour paths (e.g., circles vs. S-contours) and there existed a separate global closure mechanism underpinned not only by V1 but also higher visual areas like V4, a region responsible for the perception of an object’s shape (Anitha & Connor, 2002). Thus, it remains controversial whether the integration of closed collinear contours necessitates higher level shape perception or a top-down mechanism (Li & Li, 2015). The contention is consistent with some evidence in developmental psychophysics that infants are capable of detecting contours embedded in noise although the use of information on proximity, collinearity, and closure is seen as far beyond their capacity in the development course (Hadad, Maurer & Lewis, 2010; Hipp et al., 2014; Taylor et al., 2014). In another example, children aged 3–9 seemed to use the local proximity feature rather than global closure mechanism when detecting closed and open contours embedded in noise (Hipp et al., 2014). Nevertheless, converging evidence demonstrates that some even higher level cognitive activities may apparently contribute to contour integration through a feedback mechanism, such as learning (Li, Piëch & Gilbert, 2008), training (Lavie et al., 2009), or conscious awareness (the state of being conscious of an external object or fact) (Li & Li, 2015; Pitts, Martã-Nez & Hillyard, 2012). For example, Li, Piëch & Gilbert (2008) found that contour integration in V1 strongly depended on perceptual learning and less salient contours could be easier to detect after learning and training owing to top-down influences. Collinear but not orthogonal contours could even be processed in the absence of conscious awareness (Jimenez, Montoro & Luna, 2017; Li & Li, 2015). Moreover, an electrophysiological study indicated that ERP differences in early stages between square contours and random arrays reflected contour integration unconsciously while the difference in late stages reflected the processing of contours consciously (Pitts, Martã-Nez & Hillyard, 2012).

The problem of interest is how high-level cognitive factors, such as attention, and low level features, such as proximity, interact in visual perception. Perceptual load, a well-documented concept taxing the limited perceptual processing capacity, is known to tune attention selection. A higher perceptual load means either a larger number of items must be perceived or there will be more difficulty in perceptually processing each item. According to Lavie’s Load Theory, the level of perceptual load in a relevant task determines the extent to which irrelevant stimuli are processed (Lavie, 1995; Lavie et al., 2004). When task-relevant stimuli require a high level of perceptual load that exhausts all capacity available, task-irrelevant stimuli will be prevented from being processed (early attention). By contrast, any remaining capacity from the task-relevant processing will result in the perception of irrelevant stimuli involuntarily (late attention). Moreover, previous evidence shows that the role of the perceptual load is also tightly entangled with conscious awareness and the irrelevant stimuli automatically compete with task-relevant stimuli for the allocation of attentional resources regardless of whether or not an observer is consciously aware of them (Bahrami et al., 2008; Chen et al., 2016; Lavie, 2006; Lavie et al., 2004; Tyndall, Ragless & O’Hora, 2018). For example, another study of Lavie’s team verified that conscious awareness of task-irrelevant stimuli was decided by the level of perceptual load in task-relevant processing in a series of experiments that modified the inattentional blindness (IB) paradigm (Cartwright-Finch & Lavie, 2007). Inattentional blindness refers to the inability to report visible but unexpected objects when attention is occupied by a major task (Most et al., 2005). However, Bahrami et al. (2008) manipulated the perceptual load for a foveal cross detection task (relevant) as low load (detecting color) or high load (detecting conjunctions of color and shape) and found that the performance of an irrelevant peripheral orientation discrimination task was determined by the level of perceptual load. The authors asserted that the spill-over of spare perceptual capacity in the foveal task with low load could contribute to the unconscious processing of the peripheral orientation stimulus while the consumption of most or all of the capacity by a foveal task with high load could prevent or eliminate it. Thus, we attempted to investigate how the perceptual load influences the extent of contour processing in an irrelevant task with conscious awareness being manipulated.

This study was conducted to investigate the effect of perceptual load on contour detection and integration through saliency (different element spacing), and topological properties (circle or S shaped) with or without conscious awareness. Inspired by relevant studies, we combined a modified IB paradigm with a manipulation of the perceptual load to implement our research question (Bahrami et al., 2008; Cartwright-Finch & Lavie, 2007; Pitts, Martã-Nez & Hillyard, 2012). For example, Pitts, Martã-Nez & Hillyard (2012) adopted a modified version of the IB paradigm adapted for ERPs to identify neural activity associated with different stages of contour detection and conscious awareness. Additionally, due to the impossibility of directly measuring the processing of invisible contours in our experiment, an indirect measurement known as the Posner cuing effect was conducted (Posner, Snyder & Davidson, 1980). The Posner cuing paradigm, a well-established method to study the effects of covert orienting of attention, has been utilized recently to investigate the unconscious processing of visual stimuli such as pictures and contours (Li & Li, 2015; Yi et al., 2006; Zhang et al., 2012). For example, Zhang et al. (2012) used a modified Posner cuing paradigm to assess the degree to which the unconscious processing of orientation contrast between an invisible foreground and the background affected a subsequent localized discrimination task (e.g., a dot or Gabor probe). They found the invisible foreground induced a positive cuing effect and the effect increased with the magnitude of the orientation contrast. Notably, a more recent study has delved into the level of processing of circular contours in both conscious and unconscious conditions with a combination of the Posner cuing paradigm with a modified IB paradigm (Li & Li, 2015). The authors concluded that collinear but not orthogonal contours could be integrated without conscious awareness, leading to a positive Ponser cuing effect. Moreover, they managed to validate the conclusion with a breaking continuous flash suppression (b-CFS) paradigm, which has been increasingly employed to study the processing of various stimuli in the absence of conscious awareness (Yi et al., 2006). The current study was to expand that of Li & Li’s from three aspects. First, their research focused on only one low-level feature of contours: collinear or orthogonal. We went further by exploring the joint influence of local features (saliency) and intermediate topology (admittedly speculative) on contour integration. Second, to reduce top–down attention and create deeper state of IB, we replaced their peripheral go/no-go task with a foveal go/no-go task in which participants’ attention was diverted further away by a cross of varied color and shape. Lastly, the perceptual load was manipulated in the current study to address the intriguing question of how high-level cognitive factors (attention or conscious awareness) interact with lower level factors (saliency and typology) in contour integration.

## Materials & Methods

We adopted a modified IB paradigm in which a foveal go/no-go task diverted attention away from contours and a Posner cuing paradigm was used to assess the processing level of contours (Li & Li, 2015; Pitts, Martã-Nez & Hillyard, 2012; Zhang et al., 2012). In our design, different contours were used as spatial cues and the differences in an orientation discrimination task in terms of RT (reaction time) and accuracy at the cued or uncued location were calculated to measure the Posner cuing effect. We reasoned that the presence of irrelevant contours in a cued or uncued visual field might involuntarily interfere with the subsequent behavioral performance of a primary task (Gabor orientation discrimination), and a different level of detectability of contours could lead to differential Posner cuing effects (Li & Li, 2015; Zhang et al., 2012).

**Figure 1:**
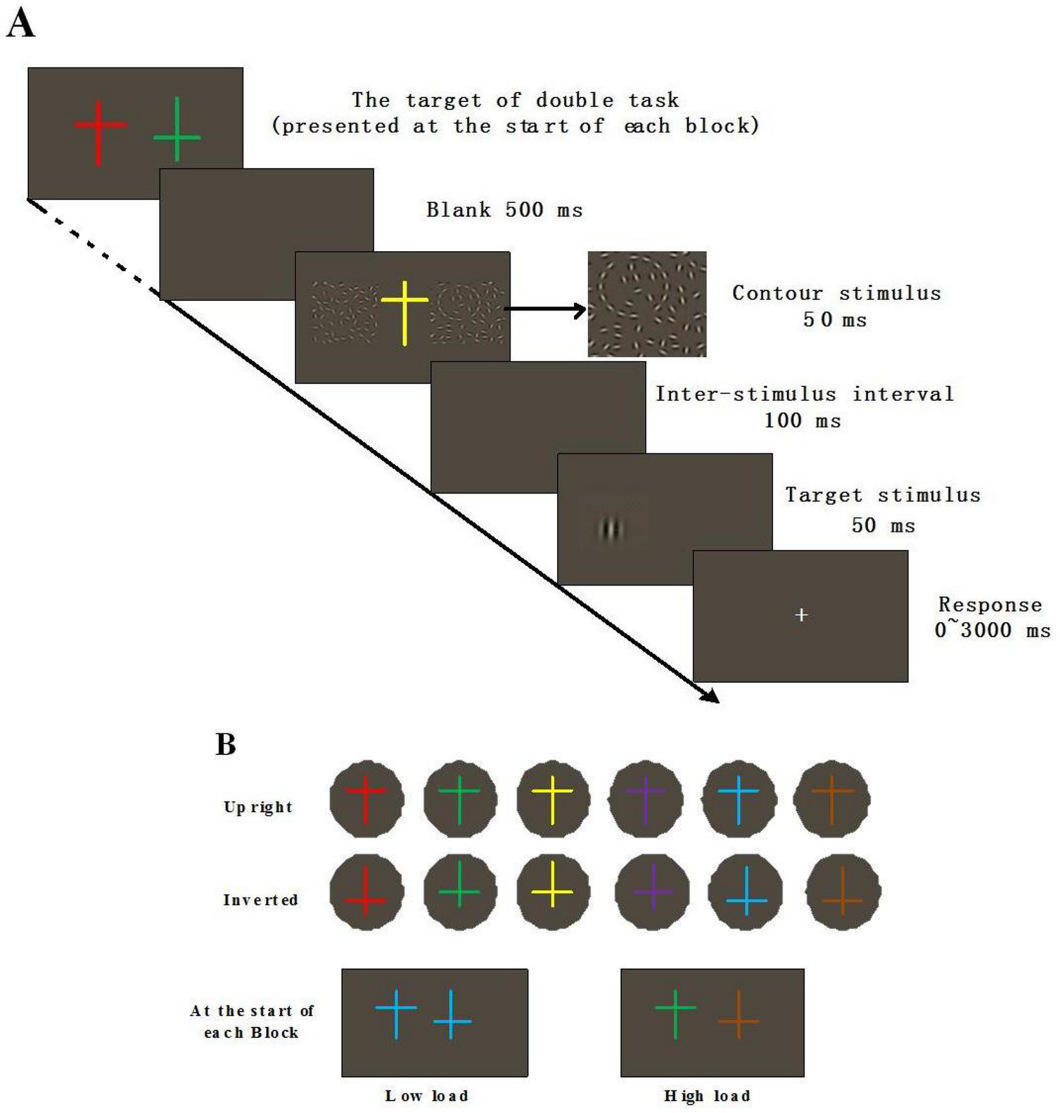
(A) Schematic illustration and (B) an example of sequences for foveal load task in Phase I and Phase III. (A) Participants were instructed to complete a dual task in which a foveal go/no-go task was combined with a peripheral Gabor orientation discrimination task with low or high perceptual load. (B) Low load, two crosses are the same in colour but different in form; high load, two crosses are different both in colour and form.

Our go/no-go task was used to direct top-down attention to the cross at the foveal, which varied in both color (including red, green, yellow, blue, purple and brown) and shape (upright or inverted), as shown in Fig. 1B. The target cross was associated with a prior memorization task in which two preceding crosses imposed cognitive loads. We reasoned that memorizing the color of the crosses, regardless of their shapes, served as low perceptual load for the go/no-go task whereas the memorization of both the color and shape of the crosses served as high perceptual load (Bahrami et al., 2008).

The experiment was separated into three phases. In Phase I, participants performed a dual task: they first saw a go/no-go target, the discrimination of which varied with perceptual load and then reported the orientation of a Gabor that was cued by four types of contours (combinations of two saliency levels and two shapes), as shown in Fig. 1A. Afterwards, participants completed a questionnaire (see Appendix) to make sure they were NOT consciously aware of the presence of the contours. To manipulate the conscious awareness of contours, participants were familiarized with the contours by performing a forced choice detection task (2AFC) on circles and S-contours in Phase II. Phase III was identical to Phase I, except that participants were consciously aware of the presence of all the contours.

Our hypotheses included that: (1) contours could not be processed under high perceptual load because of the inadequacy of attentional resources, (2) salient contours could be processed at low perceptual load with or without conscious awareness while non-salient contours could not, and (3) the processing pattern might vary between different topological properties (circle or S-contour) when perceptual load or conscious awareness were manipulated.

### Participants

Nineteen naive right-handed participants with normal or corrected-to-normal vision (7 males and 12 females), 21–27 years old (average 24 ± 1.7), participated in three phases of the experiment that lasted about two hours on two successive days with Phase I on one day and Phase II and III on the other day. Participants were provided with written informed consents beforehand and some monetary reward afterwards. The experimental paradigm was approved by the Ethics and Human Participants in Research Committee at the University of Electronic Science and Technology of China (NO.1701001) in compliance with the Declaration of Helsinki.

### Experimental setup

The visual stimuli were presented on a 21-inch color monitor with a mean luminance of approximately 22 cd/m^2^ and a frame frequency of 100 HZ at a spatial resolution of 1,280 × 1,024 pixels. The experimental program was compiled by MATLAB 2013 combined with Psychtoolbox (Brainard, 1997). Participants viewed the stimuli presented on a grey background at the distance of 57 cm with a head and chin rest, in a sound-attenuated room.

### Stimuli

#### Stimuli for go/no go task

The first kind of stimuli served as a load that required memorization at the start of each block and the second kind of stimuli served as a target that required go/no-go responses in each trial. The load stimuli were two parallel 0.5° × 0.75° crosses located in the center of the monitor that varied in color (red, green, yellow, blue, purple, or brown) or shape (upright or inverted), as Fig. 1B illustrates. The target stimulus was only one 0.5° × 0.75° colored cross at the fixation point.

#### Stimuli for Gabor orientation discrimination task

A target Gabor (peak spatial frequency: 2.5 cycles/degree, envelop *σ*: 0.27°, contrast: 130%), tilted either 1.5° clockwise or counterclockwise from the vertical meridian was used in an orientation discrimination task, as shown in Fig. 1A.

#### Stimuli as Posner spatial cues

**Figure 2:**
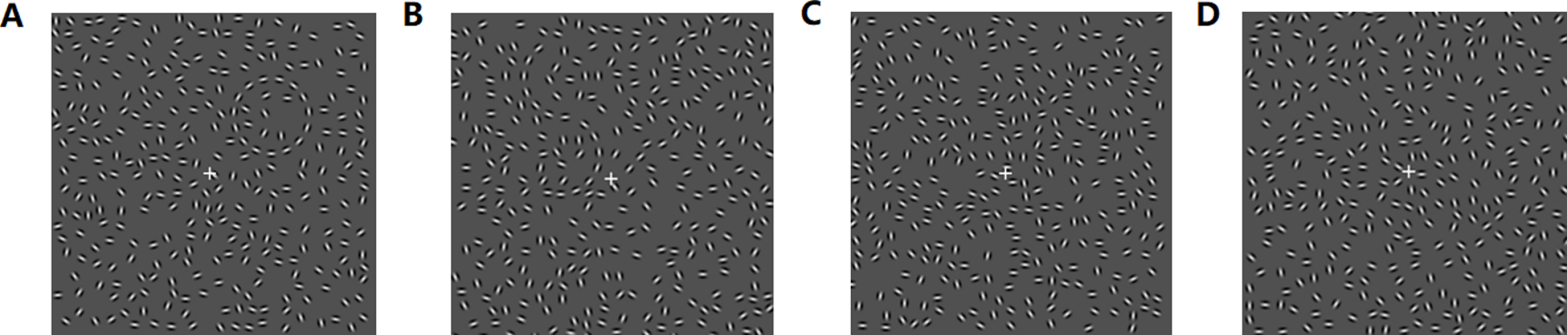
Cuing stimulus examples in Phase I, II and III. (A) Salient circle contour; (B) salient S-contour; (C) non-salient circle contour; (D) non-salient S-contour.

The saliency of contours was defined as the different distances between the adjacent contour segments and was manipulated by changing the number of Gabor elements with salient contours consisting of fifteen Gabor elements while non-salient contours consisted of only eleven elements. The topological properties of the contours were defined as circles and S-contours that were both collinear and of the same local size with a radius of 1.13°. The two kinds of contours contained the same number of elements with the same local curvature angle, as two halves of the circle could be transposed to form an S-contour. Saliency and the topological properties were combined into four types of cuing stimuli (salient circle, salient S contour, non-salient circle, and non-salient S contour), which were all embedded in the same Gabor field with a size of 12.08° × 12.08°. The Gabor field was generated on an algorithm adapted from a relevant study (Dakin & Baruch, 2009). It contained approximately 300 Gabor elements rendered randomly in odd or even numbers. All the elements were in cosine phase and had a peak spatial frequency of 2.08 cycles/degree, an envelope of 0.13°, a contrast of 130%, and an average inter-element space of 0.68°, as shown in Fig. 2.

### Procedure

#### Phase I

Before the experiment, participants did 60 practice trials without any contour in order to familiarize themselves with the procedure. Participants were instructed to make a Gabor orientation discrimination task as quickly and accurately as possible. Those whose accuracy exceeded 80% could continue with the formal experiment which consisted of eight blocks with a total of 576 trials. Contour types were randomly chosen and embedded in one of the two Gabor fields. Each type of contour appeared 14–15 times in one block of 72 trials. We manipulated the perceptual load block-by-block rather than trial-by-trial. There were four high-load blocks in which participants were required to memorize both the color and shape of the two crosses and the other four low-load blocks in which participants were required to memorize only the color of the two crosses. The block order was counter-balanced through prior experimental instructions on the screen (Bahrami et al., 2008). At the start of each block the load stimuli of two crosses were presented at the center of the monitor. The participants were asked to observe and memorize the color or the conjunctions of shape and color in two crosses without any time limit until they felt ready to start a dual task by clicking the right button on the mouse. Task one was a central go/no-go task and task two was a peripheral Gabor orientation discrimination task. Figure 1A shows the schematic illustration of one trial in each block plus the initial memorization task. After 500 ms of blank screen, a colored cross was presented for 50 ms at the fixation point with two square Gabor fields located to its right and left sides, which contained cuing contours (80% trials) or no contour (20% trials). The color and shape of the target cross was randomized within a block. After a 100 ms inter-stimulus-interval (ISI) of another blank screen, a target Gabor was presented for 50 ms, either at the location of the contour side (cued position) or at the opposite side (uncued position). The subjects were then instructed to click the mouse as accurately and quickly as possible to discern the orientation of the target Gabor when the central target cross was the same as one of the two crosses in both color and shape at the beginning of the block (go trials), and not to press any button when the central cross was the same as one of two crosses only in color (no-go trials). The background remained grey until the participants responded by clicking either the right (1.5° clockwise) or left button (1.5° counterclockwise) on the mouse to indicate the orientation of the target Gabor. The accuracy and RT (from onset of the target Gabor to response) for the response were both recorded. The next trial began immediately after a response or after three seconds without any response. Immediately after eight blocks, the participants were asked to complete a questionnaire to assess their awareness of the presence of the cueing contours (Li & Li, 2015; Pitts, Martã-Nez & Hillyard, 2012). The confidence in the detection of six types of contours and the frequency of their presences were determined on a five-point scale.

#### Phase II and III

**Figure 3:**
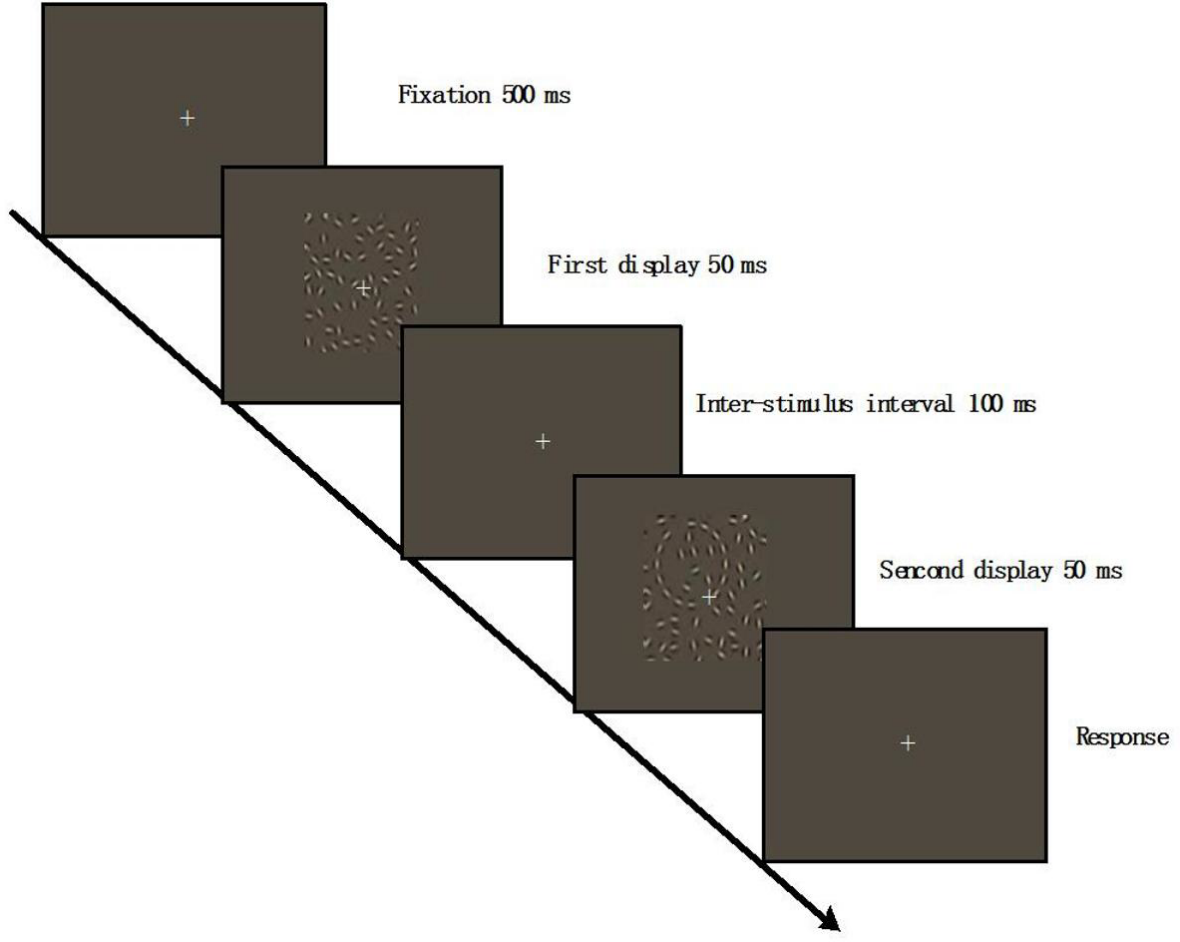
Schematic illustration of phase II. One of four contours was embedded in one of two Gabor fields. The contours appeared randomly in any region of the Gabor field. The participants were required to judge which field contained a contour as quickly and accurately as possible.

Prior to the formal blocks in phase II, participants were presented with four types of contours and clearly informed of the occurrence of contours in the coming trials. If they had difficulty in detecting any contour, the experimenter helped them by pointing it out on the screen. They were also required to familiarize themselves with the procedure in a training session consisting of 40 trials of merely salient contours. After the training, they were asked to complete a temporal two-alternative forced-choice (2AFC) contour detection task, as shown in Fig. 3.

Each trial started with a 500 ms fixation (0.4° × 0.4° ) followed by the first 12.08° × 12.08° Gabor field being presented at the center of the screen for 50 ms. After the 100 ms ISI of a fixation point sized 0.75° × 0.75°, the second Gabor field was displayed for 50 ms again. One of four contours (cueing contours used in Phase I) was embedded in one of two Gabor fields (same size in Phase I). The contours appeared randomly in any region of the Gabor field. The participants were required to judge which field contained a contour as quickly and accurately as possible. No matter whether the participants saw the contour or not, they were forced to make a response. The background remained gray until the participant made a button press in 3 s. Each participant completed 3 blocks of the detection task, consisting of 144 trials with 12 trials for each contour type. The trials of four contour types were randomly ordered.

This phase was expected to evaluate the detectability of different types of contour without a perceptual load and to reinforce the participants’ conscious awareness of all cueing contours, serving as a control study for Phase I and the pilot study for Phase III, as shown in Fig. 1A. Presumably, the only difference between Phase III and Phase I was whether or not the participants were conscious of the presence of the contours.

## Results

### Awareness questionnaires of Phase I and III

It is believed that, due to the manipulation of perceptual load in our experimental design, attention allocated to contour detection in both Phase I and III was further reduced. Nineteen participants completed all three phases and filled out two questionnaires. According to the awareness questionnaires, 11 out of 19 participants were not consciously aware of circle contours, while 15 were not consciously aware of S-contours in Phase I since they rated their confidence level around a 2 (2: “confident I didn’t see any shape of contours”). Nineteen participants reported seeing both types of contours and rated their confidence level as 4 or more in Phase III (4: “confident I saw a contour”). Their average scores and standard errors for both awareness questionnaires and the four types of contours are shown in Table 1.

**Table 1:** Results of awareness questionnaires in Phase I and III. Means and standard deviations (in parentheses) of the rating scores for circle and S-contour in the five-point awareness questionnaire. Phase I refers to the unconscious stage and phase III refers to the conscious stage. The awareness questionnaire adopted five-point scale ranging from 1 (least confident or frequent) to 5 (most confident or frequent).

Phase	Confidence		Frequency	
	Circle contour	S-contour	Circle contour	S-contour
Phase I	2.00(0.30)	2.13(0.26)	1.55(0.25)	1.40(0.19)
Phase III	4.53(0.27)	4.40(0.29)	3.53(0.31)	3.27(0.30)

The aim of this study was to investigate the processing level of contours that varied in saliency and topology in both conscious and unconscious conditions. The participants who reported not seeing any shape and rated their confidence level in seeing any type of contours as 3 or less were considered to be in inattentional blindness or in an unconscious state in Phase I. We only analyzed the data from the fifteen participants, whose results were valid. A two-way repeated-measure analysis of variance (ANOVA)(conscious awareness [unconscious, conscious] × contour type [circle, “S”]) on confidence ratings indicated a significant main effect of conscious awareness, *F*(1, 14) = 67.045, *p* < 0.001, but the main effect of the contour type (*p* = 0.909) and the interaction between the two factors (*p* = 0.567) were not significant, as shown in Table 1. A two-way repeated-measure ANOVA (conscious awareness [unconscious, conscious] × contour type [circle, S]) on the frequency rating also revealed a significant main effect of conscious awareness, *F*(1, 14) = 44.895, *p* < 0.001, but the main effect of the contour type (*p* = 0.544) and the interaction effect (*p* = 0.741) was not significant. These results confirmed that participants were indeed in different states of conscious awareness on contour detection in the left or right Gabor field in Phases I and III, just as we designed.

### Results of central go/no-go task in Phases I and III

It is highly likely that the high-load task consumed more attention than low-load task because the attention allocated to the peripheral orientation discrimination task was reduced by the central go/no-go task (Lavie et al., 2004; Li & Li, 2015; Pitts, Martã-Nez & Hillyard, 2012). The miss rate (the number of trials where no response was given to a go-task divided by the total number of cued go trials) and false alarm (the number of trials where a response was given to a no-go task divided by the total number of cued no-go trials) were used to evaluate participants’ engagement with the task in the experiment. The data points with a miss rate higher than 10% were excluded from analysis (Bahrami et al., 2008). It turned out that the average miss rates and false alarms were 0.21%, 0.24%, and 0.17%, 0.13% for Phase I and III, respectively. Separate ANOVAs showed there was no significant difference of miss rates (*p* = 0.323) or false alarms (*p* = 0.542). The results indicated that participants were fully engaged in the experiment, laying a solid foundation for further analysis.

### Peripheral orientation discrimination performance in Phase I

**Figure 4:**
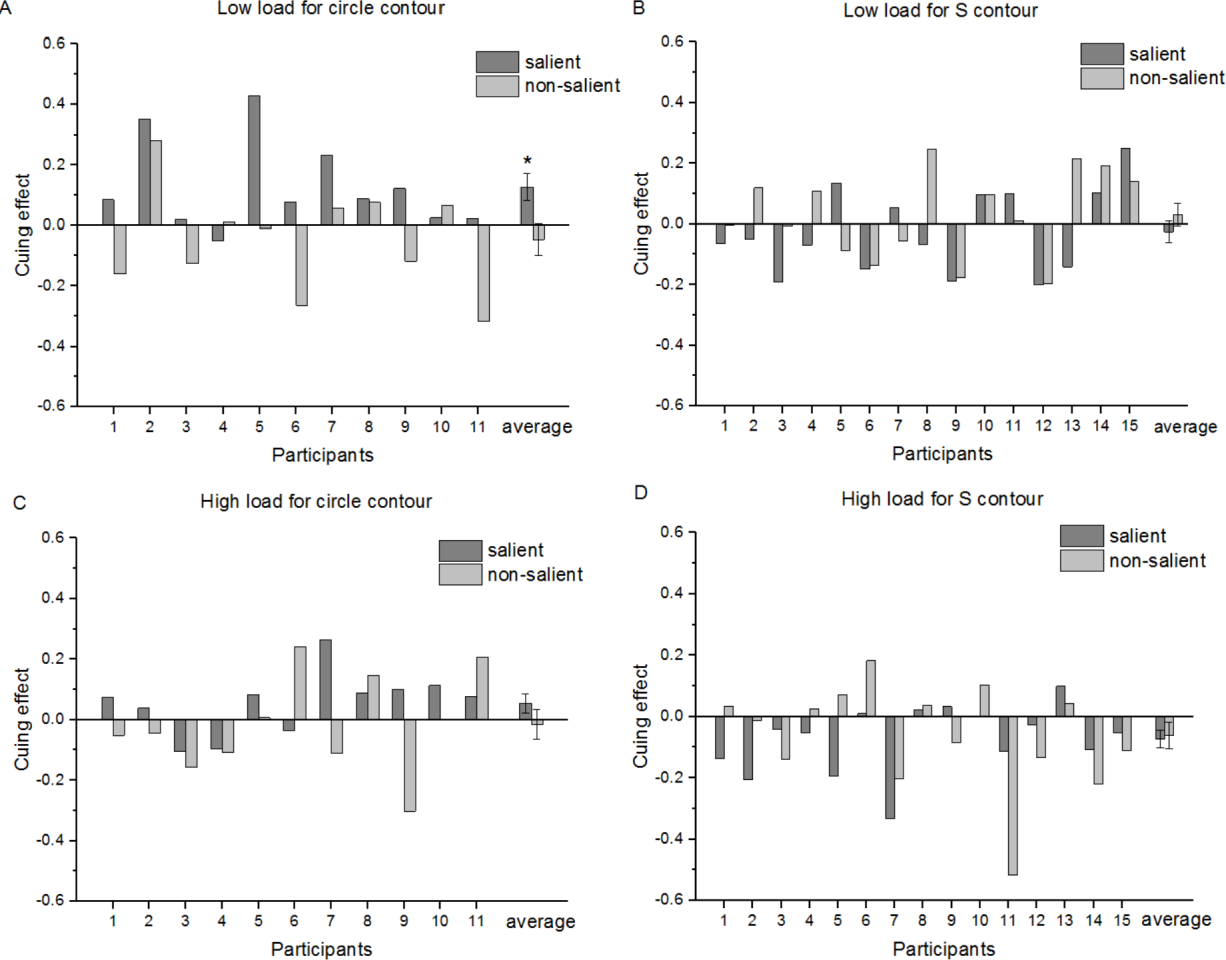
Attentional cuing effects for accuracy in an Orientation Discrimination Task between the cued and uncued conditions for each participant and the group average in Phase I. (A) Low load for circle contour; (B) low load for S-contour; (C) high load for circle contour; (D) high load for S-contour. Error bars denote SEM calculated across participants. *p < 0.05.

As mentioned above, 11 out of 19 participants were unaware of circle contours and 15 were unaware of S-contours in Phase I. Only 11 participants’ data for circle contours and 15 participants’ data for S-contours in the unconscious state were considered to be valid. Cuing effects were assessed by the difference in accuracy of orientation discrimination between the cued and uncued conditions. Since the number of participants with valid data was different between circles and S-contours, we could not use repeated measures but used a three-way ANOVA (perceptual load [high, low] × Saliency [salient, non-salient] × Contour type [circle, “S”]) (Type III SS) on cuing effects instead. Box plots showed there was no abnormal value of more than three times the box length in the data of this phase. According to the Shapiro–Wilk Test, cuing effects in all conditions followed normal distribution except for one condition (high load, non-salient, S-contour, *p* = 0.021). Levene’s Test showed homogeneity of variance (*p* = 0.679) for all conditions in the data. No significant interaction was found for the three factors or two factors (Ps > 0.05), except that a significant interaction was found between the saliency and contour shape, F (1, 96) = 8.433, *p* = 0.005. Comparative analysis demonstrated that a significant difference of the cueing effect was observed between circles and S-contours only when they were salient under a low perceptual load ($\underline{M}=0.166\pm 0.057$, *p* = 0.004 < 0.05) and there was no significant difference between both types of contours when they were non-salient under a high perceptual load ((Ps >0.05)). Post-hoc Paired-*T* tests demonstrated a significant positive cuing effect for salient circle contours ($\underline{M}=0.13\pm 0.15$, *t*(10) = 2.823, *p* = 0.018 < 0.05), but no significant effect for salient S-contours under low perceptual load ($\underline{M}=-0.03\pm 0.14$, *t*(14) =  − 1.105, *p* = 0.288), as shown in Figs. 4A and 4B.

However, if cuing effects were indexed by the RT difference in orientation discrimination between the cued and uncued conditions, no significant main effect or interaction was found in any condition (ps > 0.05). These results indicated that there was no trade-off between RT and accuracy.

### Peripheral orientation discrimination performance in Phase III

**Figure 5:**
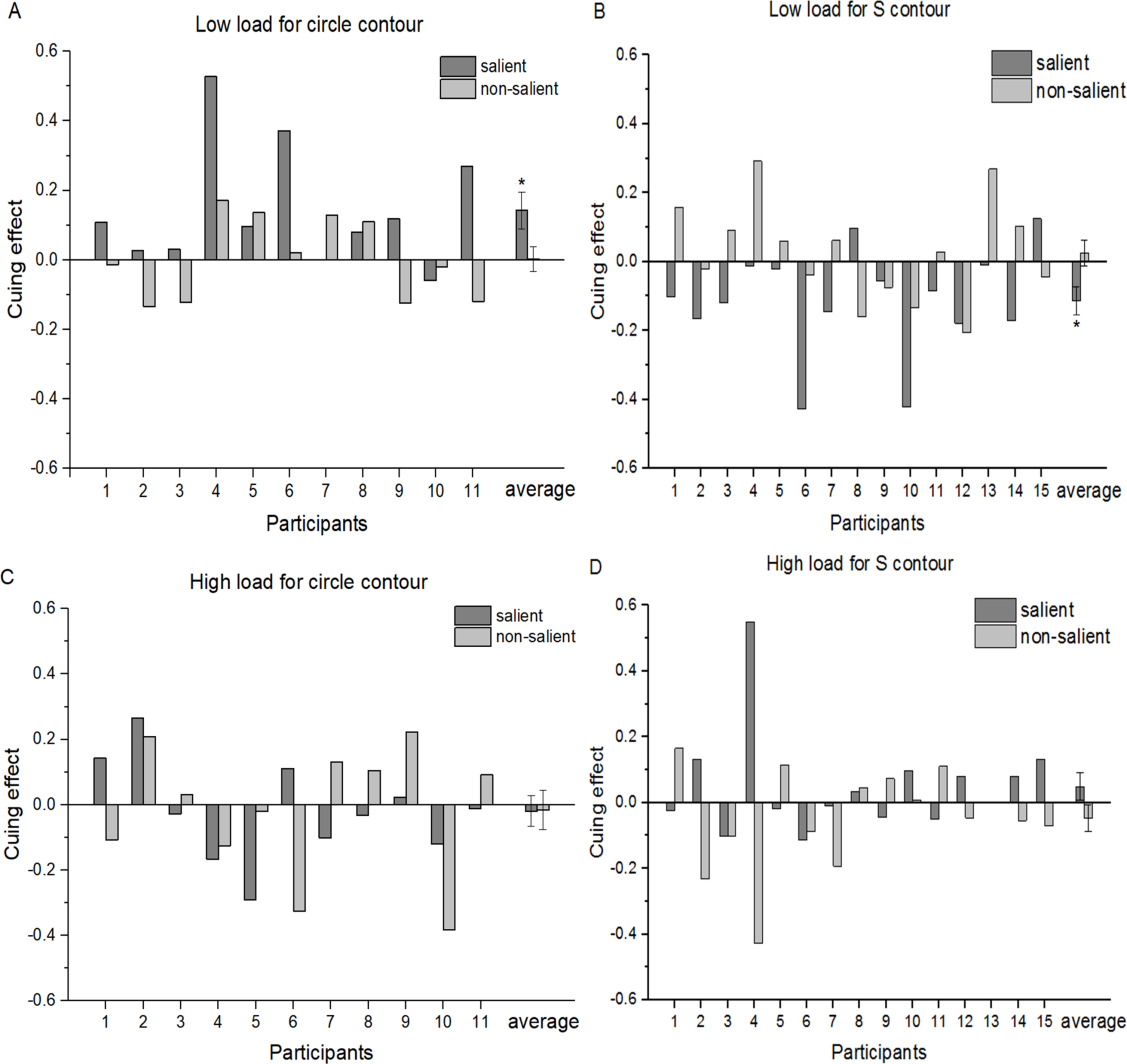
Attentional cuing effects for accuracy in the Orientation Discrimination task between cued and uncued conditions for each participant and a group average in Phase III. (A) Low load for circle contour; (B) low load for S-contour; (C) high load for circle contour; (D) high load for S-contour. Error bars denote SEM calculated across participants. *p < 0.05.

As mentioned above, the goal of this study was to compare the detectability of four different contours under either conscious or unconscious conditions. As in Phase I, a three-way ANOVA (Type III SS) was used to analyze the effects of perceptual load, saliency, and contour shapes on cuing effects. According to the box plot, there was an abnormal value of more than three times the box length in the data (high load, salient S-contour, cuing effect 0.55). Shapiro–Wilk Test indicated that the data in all conditions followed a normal distribution (Ps > 0.05) except one condition (high load, salient, S-contour, *p* = 0.001). Levene’s Test confirmed that homogeneity of variance (*p* = 0.871 > 0.05) existed for all the conditions. The results revealed that there was a significant interaction of the three factors, *F*(1, 96) = 8.968, *p* = 0.003. No significant interaction was found between two factors (Ps > 0.05) except for a significant interaction between perceptual load and contour shape, *F*(1, 96) = 4.625, *p* = 0.034. Simple effect analyses indicated that there was a significant difference between two contour shapes when they were presented under low perceptual load, *F*(1, 96) = 6.874, *p* = 0.01 < 0.05, but none when presented under high load, *F*(1, 96) = 0.176, *p* = 0.676 > 0.05. Comparative analysis showed that there was a significant difference between circles and S-contours only when they are presented saliently under low perceptual load ($\underline{M}=0.256\pm 0.063$, *p* = 0.000 < 0.001) and no significant difference under high perceptual load ($\underline{M}=-0.068\pm 0.063$, *p* = 0.280 > 0.05). Notably, if we excluded the abnormal value mentioned above, the pattern of ANOVA results remained similar to that of the results when the outlier was included. Post-hoc Paired-*T* tests showed a significant positive cuing effect for salient circles ($\underline{M}=0.108\pm 0.177$, *t*(10) = 2.678, *p* = 0.023 < 0.05), but a negative cuing effect for salient S-contours when presented under low perceptual load ($\underline{M}=-0.113\pm 0.156$, *t*(14) =  − 2.808, *p* = 0.014 < 0.05), as shown in Figs. 5A and 5B.

However, if cuing effects were indexed by the RT difference in orientation discrimination task between cued and uncued conditions, no significant main effect or interaction was found in any condition (*p* > 0.05). These results also indicated that there was no tradeoff between RT and accuracy.

Taken together, it was only in low load conditions that circles rather than S-contours induced an positive cueing effect unconsciously while they showed opposite patterns consciously (circle: positive; S-contour: negative). These results were applicable only when the contours were salient.

### Contour detection performance in Phase II

**Figure 6:**
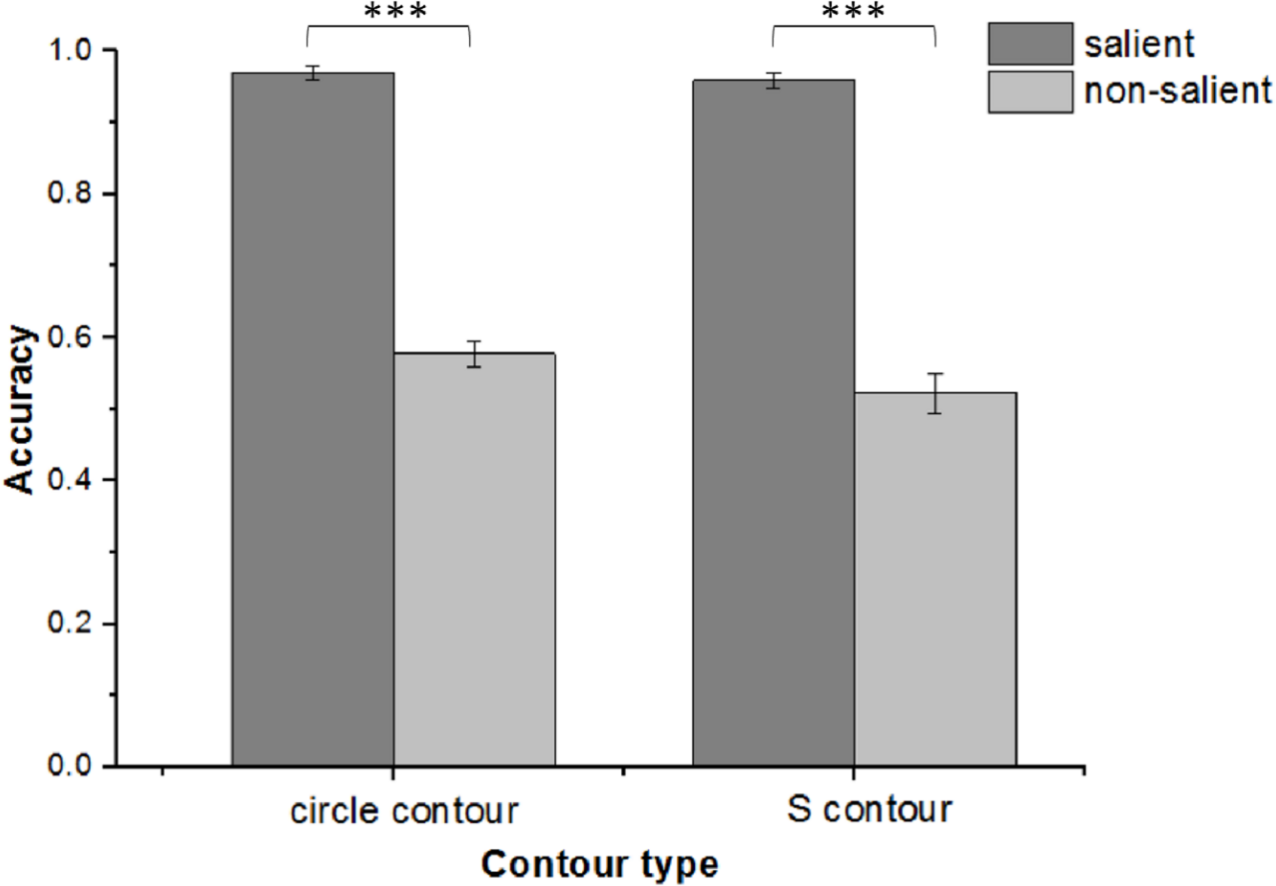
Accuracy performance in Phase II . Accuracy performance for salient/non-salient circles, salient/non-salient S-contours in Phase II. Error bars denote SEM calculated across participants. ***p < 0.001.

The performance of accuracy in Phase II is shown in Fig. 6. As mentioned above, here we tried to assess the detectability of different types of contours with sufficient conscious awareness after the participants familiarized themselves with both the stimuli and procedure in advance. Data from 15 participants was included in the analysis to maintain consistency between Phases I and III. A two-way repeated-measure ANOVA (topological property [circle, “S”] × saliency [salient, non-salient]) for the 2AFC task revealed a non-significant main effect of topological property, *F*(1, 14) = 4.54, *p* = 0.053, a significant main effect of saliency, F(1, 14) = 480.646, *p* < 0.001, and a non-significant interaction between the two factors, *F*(1, 14) = 1.269, *p* = 0.279. Thus, the detection performance for salient contours was much better than that for non-salient contours with the latter having an accuracy of a little above chance (M_salient circle_ = 0.97 ± 0.04, M_salient s-contour_ = 0.96 ± 0.04; M_non-salient circle_ = 0.58 ± 0.07, M_non-salient s-contour_ = 0.52 ± 0.1). Notably, there was no significant difference in the accuracy of detecting circles and S-contours whether they are salient or not.

In addition, RT data was also analyzed. Because there was no time limit for subjects to respond, the data for a few trials was too great to be included in overall analysis. Any data point more than two standard deviations above average in each condition was considered an outlier and excluded from the final analysis. The elimination rate of data from four conditions was 1.48%, 5.74%, 1.11%, and 8.33%, respectively. The remaining data underwent the same two-way repeated-measure ANOVA and the results were consistent with those reported for accuracy. There was no significant main effect for topological property, *F*(1, 14) = 4.47, *p* = 0.053, but a significant main effect for saliency, *F*(1, 14) = 38.29, *p* < 0.001, and no significant interaction between the two factors, *F*(1, 14) = 0.097, *p* = 0.76. Thus, the detection for salient contours was much quicker than that for non-salient contours whereas there was no significant difference between circles and S-contours whether they were salient or not (M_salient circle_ = 0.44 ± 0.09 s, M_salient s-contour_ = 0.47 ± 0.11 s; M_non-salient circle_ = 0.69 ± 0.19 s, M_non-salient s-contour_ = 0.70 ± 0.18 s). In summary, the detection of salient contours was faster and more accurate than that of non-salient contours when no perceptual load was involved. However, topologically, there was no significant difference in detectability between the circles and S-contours.

## Discussion

The findings from the three-phase experiment conformed to all the research hypotheses except the null findings on RTs. Based on the statistics of the cueing effect on accuracy of the orientation discrimination task, we obtained three major results. First, either salient circles or salient S-contours induced a consistent pattern of Posner cuing effects under low perceptual load while neither type could induce any obvious pattern under high perceptual load. Second, no significant pattern of cuing effects was found for both non-salient contours whether under high or low perceptual load. Third, under low perceptual load, different patterns of cuing effects were found between salient circles and salient S-contours. Specifically, a positive cuing effect (facilitation) was found for salient circles both consciously and unconsciously while a negative cuing effect (inhibition) occurred for salient S-contours only in the conscious state.

First, the results have been predicted by the Load Theory (Bahrami et al., 2008; Lavie, 2006; Lavie et al., 2004). In our experiment we manipulated the perceptual load by combining an initial load-based memorization task with a subsequent foveal go/no-go task, which resulted in the change of the level of inattentional blindness (IB) or the state of conscious awareness. We found, when the foveal task involved low perceptual load rather than high load, salient circles and S-contours produced different cuing effects. It was inferred that typological properties could be processed under low perceptual load. Given the finding of the same detectability in Phase II involving no perception load, we contended that perceptual load did modulate topological properties in contour integration. Our arguments were presented as followed: tasks with high perceptual load (detecting the conjunctions of color and shape) could engage full attentional capacity leaving no capacity for the processing of any contour, which may explain the null findings for either type of contours with high perceptual load whether they were presented saliently or not. However, as long as the go/no-go task imposed only a low level of perceptual load (detecting color) on attention, the spared attentional resource could be utilized to process irrelevant contour stimuli in terms of topology either automatically (unconsciously) or intentionally (consciously). Remarkably, even if unexpected and invisible in Phase I, salient circles could also be processed or integrated unconsciously. This result is in accord with one recent study which reported implicit perception of topological properties of “holes” under b-CFS (Chen, Zhou & Chen, 2014b). Note that the b-CFS paradigm is known as a more sensitive way to investigate unconscious processing based on interocular suppression. Moreover, the electrophysiological study using the IB paradigm confirmed that contours could be integrated even if they were task-irrelevant and consciously unperceived due to the evidence that an integration-related negative component was observed in the occipital area between 220 and 260 ms after stimulus onset (Pitts, Martã-Nez & Hillyard, 2012).

Second, prior studies have shown that the processing of contours was influenced by element scale and phase (Hess, Hayes & Field, 2003), element spacing (Kovács & Julesz, 1993; Li & Gilbert, 2002) and element orientation (Li & Li, 2015; Nelson & Frost, 1985). In our experiment, we changed the number of elements in a fixed-size contour to manipulate salience levels as salient or non-salient. Our findings showed that salient contours could induce obvious patterns of cuing effects on accuracy with low perceptual load, consciously or unconsciously, but non-salient contours couldn’t. However, in the control study, non-salient contours still had an above-chance accuracy (M_circle_ = 0.58 ± 0.07, M_s-contour_ = 0.52 ± 0.1) with full attention. It could be reconciled that visual saliency was a fundamental feature in contour detection. That is, the saliency of contours could enjoy privileged but coarse perceptual processing as long as it reaches a certain threshold. It was hypothesized that the detection of contour saliency was mainly a bottom-up process driven by the low-level features of visual stimuli such that it was less sensitive to the modulation of top-down attention when the salience level remained underneath the threshold (Li, 1997; Zhang et al., 2012). This hypothesis was reminiscent of the previous findings that the performance of detecting salient contours was better than that of non-salient contours (Li & Gilbert, 2002; Vancleef & Wagemans, 2013). This also conformed with both the psychophysical (cuing effect) and physiological (ERP component and fMRI signal) evidence from a comprehensive study, which confirmed that V1 was the neural substrate of saliency that tuned only to primitive features rather than complex objects even if they were invisible to observers (Zhang et al., 2012). Therefore, we could not draw a hasty conclusion that contour saliency was not affected by the perception load since the display time in our experiment was only 50 ms, which might temporally result in lack of feedback information from the higher visual cortex. It is more likely that a longer display time or more training is required to make it easier to distinguish non-salient contours from the cluttered background (Li, Piëch & Gilbert, 2008).

One of our interests was to identify whether a closure effect was found in contour integration when conscious awareness was manipulated. The critical manipulation was the inclusion of Phase II where the participants were provided with enough training of contour types. The training guaranteed that participants performed the task with sufficient conscious awareness in Phase III, which made our manipulation of awareness possible. When we compared Phase I with Phase III, we found the facilitating effect (positive cuing) occurred for circles both consciously and unconsciously while an inhibiting effect (negative cuing) occurred for S-contours only consciously in low load conditions. This finding suggested that closed but not open contours could be successfully integrated in an absence of conscious awareness. Thus, it could be assumed that there existed some kind of closure effect or advantage in an unconscious state. However, it was difficult to determine its existence in a conscious state under low perceptual load due to the opposite patterns of the cuing effect for two contour types in the current study, although we believed both types could be successfully integrated under those conditions. Some prior evidence demonstrated that visual integration of contour elements was more effective for closed contours than open contours due to a top-down global mechanism in a conscious state (Gerhardstein et al., 2012; Kovács & Julesz, 1993; Sweeny, Grabowecky & Suzuki, 2011). Thus, the discrepancy seemed to echo the question of whether the closure effect could be attributable to a separate global mechanism (feedback) or local feed-forward mechanism (Hipp et al., 2014; Li & Li, 2015; Tversky, Geisler & Perry, 2004). Our finding might be interpreted as the combination of bottom-up and top-down mechanisms by which information is efficiently processed in the brain (Li & Li, 2015) and conscious awareness had a pivotal role to play in coordinating when and how the bi-directional mechanism worked out. It was likely that the closure effects in unconscious states were due to the bottom-up hypothesis that the interaction of some local mechanisms (not confined to continuation or proximity) contributed to the detection advantage of circles over S-contours (Tversky, Geisler & Perry, 2004). However, when visual processing is done consciously, the closure effect might not be ubiquitous. The task and expectation were also known to greatly influence visual processing through top-down mechanism, which could account for the uncertainty or blurring of the closure effect in Phase III (Gilbert & Li, 2013; Li, Piëch & Gilbert, 2008). It seemed plausible that the weak closure effect in the feed-forward sweep was submerged by the feedback information when contours were both familiar and expected due to adequate training in the current study. Some imaging studies also confirmed that low level feed-forward information from the primary visual cortex (V1) and high level information from higher cortical areas (i.e., V4 and lateral occipital regions) co-regulated the processing of contours (Anitha & Connor, 2002; Chen et al., 2014a; Shpaner et al., 2013).

Moreover, the different patterns of cuing effects for circles and S-contours, consciously or unconsciously, under low perceptual load might also be interpreted with two well-known mechanisms in the visual processing field. The first one is called the Topological Perception Mechanism or “Global First”, which supports the functional hierarchy of “Global to Local” instead of “Local to Global” in visual perception (Chen, 2005). Based on this mechanism, Chen and his team asserted that topological properties had a high priority in human visual processing, and the number of “holes” had a greater chance of being detected even in the absence of conscious awareness, which suggested the possible subcortical visual processing of topological properties (Chen, Zhou & Chen, 2014b). Thus, in our experiment, a circle including one hole was supposed to have an advantage over the S-contour without any hole, resulting in better detectability and facilitation in the unconscious state. Meanwhile, this mechanism could also account for the negative cuing effect (inhibition) for S-contours observed in the conscious state. The difference in global visual forms might give rise to negative cuing effects although two types of contours had the same number of elements with the same local curvature angle. We speculated that the implicit processing of the S-contours whose two halves were open upside to the right and downside to the left might interfere with the subsequent explicit orientation discrimination of Gabors (tilt of either 1.5° clockwise or counterclockwise), leading to the negative cuing effect. The second well-documented mechanism is called figure-ground segmentation. Kovács & Julesz (1993) assumed that closure served as a geometric heuristic useful for figure-ground segmentation which occurred at the contour boundary separating two regions, one side might be seen as the “inside” with shape and the other might be the “outside” without any shape (Gerhardstein et al., 2012). Thus, an S-contour with two different curvatures might lead to inconsistent figure-ground segmentation, which could explain the negative cuing effect in the conscious state. Specifically, the top half suggested that the inside was to the right of the contour while the bottom half suggested the inside was to the left of the contour (Gerhardstein et al., 2012). In our experiment, the different figure-ground assignment, biased by each half of an S-contour, coupled with the manipulation of perceptual load, might add to the difficulty of detection and cause some kind of inhibition of return (IOR) in cued conditions of the orientation discrimination task. Taken together, more sophisticated visual forms besides circles and S-shaped contours need to be probed in the future to demonstrate the mechanisms of topological perception and figure-ground segmentation implicated in contour integration.

Lastly, the closure effect in unconscious states under low perceptual load in our experiment deserved more discussion since much controversy existed in the long history of relevant studies on “unconsciousness”. Sweeny, Grabowecky & Suzuki (2011) found that the global processing of closed curvatures couldn’t be accomplished in terms of pattern aftereffects in a CFS state. This finding also seemed at odds with our finding. The inconsistency might be caused by variations in experimental design and different definitions of “unconscious”. According to the Global Neuronal Workspace theory, unconsciousness has two very distinct types known as subliminal and preconscious (Dehaene et al., 2006). Subliminal unconsciousness is a condition where the bottom-up activation, induced by stimuli itself, is insufficient to trigger large-scale reverberation in masking or the CFS paradigm, whereas preconsciousness can be caused by lack of top-down amplifications of neurons in IB or Attentional Blink paradigm (Kouider & Dehaene, 2007). Accordingly, our findings fit better with the preconscious type rather than the subliminal type. However, one limitation of our study is that we only used seemingly subjective awareness questionnaires to verify unconsciousness. In the future we need to combine the b-CFS paradigm with ERP components, which may be more sensitive to unconscious processing (Pitts, Martã-Nez & Hillyard, 2012).

## Conclusions

This study demonstrated how the perceptual load interacted with saliency and topological properties in affecting contour integration with or without conscious awareness. Successful contour integration could be made possible only under low perceptual load rather than high perceptual load. Saliency was mainly a primitive and feed-forward feature of contour detection with minimal influence from the perceptual load. Conscious awareness played a pivotal role in coordinating a closure effect with the level of perceptual load. It was under low perceptual load that salient circles but not S-contours could be successfully integrated unconsciously, although both could be done consciously. These findings provide further evidence for contour integration through a bi-directional mechanism that low-level sensory features may interact with high-level cognitive factors. Future work is needed to pinpoint the neural underpinnings of this mechanism.

##  Supplemental Information

10.7717/peerj.7550/supp-1 Supplemental Information 1 Data for results in Phases I, II, and III There are four working sheets for Table 1, Figs. 4–6 and RTs in Phase II.

10.7717/peerj.7550/supp-2 Supplemental Information 2 Raw data for three phases The two major files for Phases I, II, and III, containing the raw data for all participants.

10.7717/peerj.7550/supp-3 Supplemental Information 3 Awareness Questionaire Awareness Questionaire includes four questions and six types of contours.

## Additional Information and Declarations

### Data Availability

The following information was supplied regarding data availability: The raw data are available as Supplemental File which consists of the experimental programs, materials and results in three phases.
