# Supplementary material for: Perceptual load modulates contour integration in conscious and unconscious states
Source: PeerJ. 2019 Aug 22;7:e7550. doi: 10.7717/peerj.7550 (PMC6708573; doi:10.7717/peerj.7550)
Supplement: Supplemental Information 3 — Awareness Questionaire includes four questions and six types of contours. [file peerj-07-7550-s003.docx]

**Appendix**

**Awareness questionnaire**

**Phase： Participant’s Num：**

1. During the experiment，did you see any form of contours？
2. If you see the contour，please describe it in detail.
3. How confident are you that you saw the forms of contour shown in the screen？

| Forms of contour | Very confident  (I didn’t see it) | Confident  (I didn’t see it) | Uncertain | Confident(I saw it) | Very confident  (I saw it) |
| --- | --- | --- | --- | --- | --- |
| ① | 1 | 2 | 3 | 4 | 5 |
| ② | 1 | 2 | 3 | 4 | 5 |
| ③ | 1 | 2 | 3 | 4 | 5 |
| ④ | 1 | 2 | 3 | 4 | 5 |
| ⑤ | 1 | 2 | 3 | 4 | 5 |
| ⑥ | 1 | 2 | 3 | 4 | 5 |

1. How often did you see the forms of contour in the screen？

| Forms of contour | Never | Rarely  (≤ 10times) | Infrequently  (10~50times) | Frequently  (50~100times) | Very frequently  (≥ 100 times) |
| --- | --- | --- | --- | --- | --- |
| ① | 1 | 2 | 3 | 4 | 5 |
| ② | 1 | 2 | 3 | 4 | 5 |
| ③ | 1 | 2 | 3 | 4 | 5 |
| ④ | 1 | 2 | 3 | 4 | 5 |
| ⑤ | 1 | 2 | 3 | 4 | 5 |
| ⑥ | 1 | 2 | 3 | 4 | 5 |


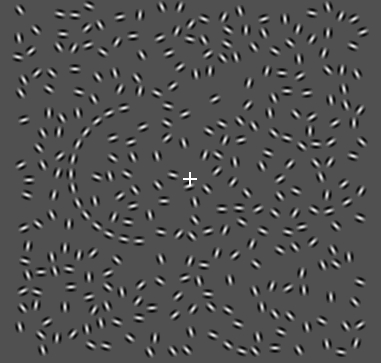

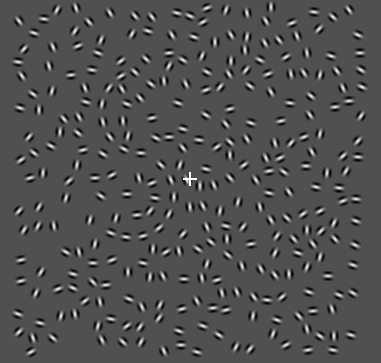


① ②


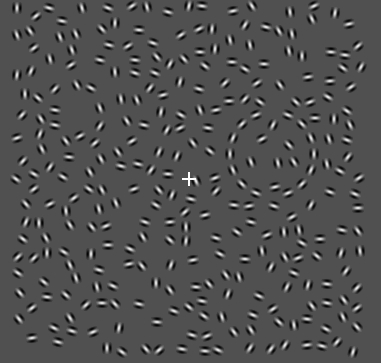

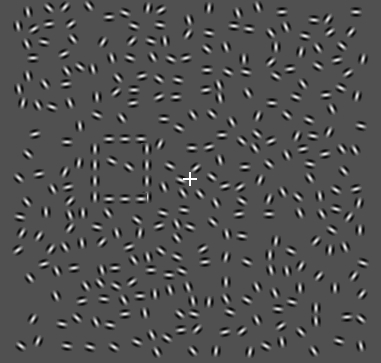


③ ④


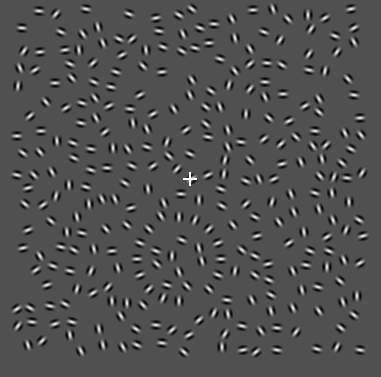

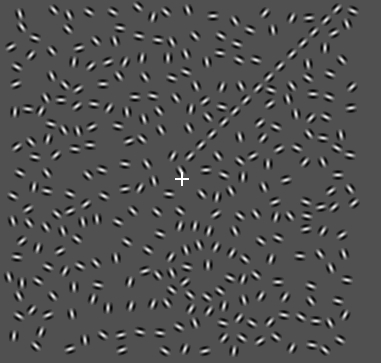


⑤ ⑥
